# A balanced review of the status T cell-based therapy against cancer

**DOI:** 10.1186/1479-5876-3-16

**Published:** 2005-04-14

**Authors:** Francesco M Marincola

**Affiliations:** 1 Immunogenetics Section, Department of Transfusion Medicine, Clinical Center, National Institutes of Health, Bethesda, Maryland, 20892, USA

## Abstract

A recent commentary stirred intense controversy over the status of anti-cancer immunotherapy. The commentary suggested moving beyond current anti-cancer vaccines since active-specific immunization failed to match expectations toward a more aggressive approach involving the adoptive transfer of in vitro expanded tumor antigen-specific T cells. Although the same authors clarified their position in response to others' rebuttal more discussion needs to be devoted to the current status of T cell-based anti-cancer therapy. The accompanying publications review the status of adoptive transfer of cancer vaccines on one hand and active-specific immunization on the other. Hopefully, reading these articles will offer a balanced view of the current status of antigen-specific ant-cancer therapies and suggest future strategies to foster unified efforts to complement either approach with the other according to specific biological principles.

A recent commentary stirred intense controversy over the status of anti-cancer immunotherapy. The commentary suggested moving beyond current anti-cancer vaccines since active-specific immunization failed to match expectations [1]. Although subsequently mitigated [2-4] the statement requires clarifications.

In the commentary the adoptive transfer of tumor antigens-specific T cells was promoted in alternative to active-specific immunization as if the two modalities were distinct biological entities rather than two extreme manifestations of the same phenomenon [1]. In fact, both exploit acquired cellular immunity in recognition of autologous cancer cells. Active-specific immunization consistently induces increments in circulating tumor antigen-specific T cell *in vivo *that are rarely sufficient to induced cancer regression [5]. We have recently argued that this observation does not represent a failure of immunization but simply a successful first step among those required to induce cancer regression [6]. In particular, circulating immunization-induced T cells physiologically display a quiescent phenotype close to memory C8+ T cells and require antigen recall plus co-stimulation in the target tissue to develop into activate effector cell [7]. Adoptive transfer of tumor antigen-specific T cells combined with nonmyeloablative chemotherapy and the systemic administration of interleukin-2 relies in the administration of *ex vivo *activated T cells and appears to be associated with enhanced clinical response rates at least in the subgroup of patients in which successful expansion is possible [1,8]. Interestingly, it was recently reported that persistence of adoptively transferred lymphocyte clonotypes significantly correlates with cancer regression [9]. This finding suggests that either *ex vivo *manipulation or *in vivo *conditions in different patients may differentially influence T cell persistence and, possibly, function. Interestingly, however, T cell persistence in itself does not clearly explain therapeutic effectiveness since other *ex vivo *expansion protocols are associated with long term persistence of adoptively transferred T cells which is not associated as tightly with tumor regression as discussed by the accompanying Yee's manuscript. In between these two extreme treatment modalities lies the combination of active-specific immunization with the systemic administration of interleukin-2 that has been associated with intermediate clinical response rates [10]. Although in the absence of prospective randomized studies it remains to be confirmed whether active-specific immunization adds to the systemic administration of interleukin-2 alone, it is reasonable to conceive the working hypothesis that the mere presence of circulating, cancer-specific T cells is not sufficient to induce cancer regression and other factors need to be added to induce their *in vivo *activation as an alternative to the *ex vivo *activation utilized for adoptive transfer. It is, therefore, our conclusion that the dichotomy between the two strategies is quite arbitrary and is merely symptomatic of our limited understanding of the requirement for effective T cell localization and activation in target tissues [6].

The surprising conclusion that anti-cancer immunization failed to yield the results anticipated by pre-clinical and early clinical studies [1,10-12] may be unduly pessimistic [3]. However, the strongest argument against a premature disposal of active-specific immunization is the recognition that our discontent comes from the naive haste in which clinical protocols have been designed in the previous decade looking primarily at clinical end-points and by-passing the more realistic goal of understanding the biology of the immune response under those conditions [6]. Rather than discarding the impressive results obtained by immunization, we should recite an introspective *mea culpa *for our failure to pay enough attention to the process of immunization at the systemic as well as at the tumor level [5]. In fairness, this negligence is due to the extreme difficulty that clinical scientists confront when studying human material and, possibly, most questions related to the understanding of human tumor immune responsiveness remain answered simply because it is exceedingly difficult to analyze tumor/host interactions in the tumor-microenvironment [13]. We have recently summarized novel strategies that could be applied to by-pass such difficulty [14-16].

In the accompanying Point-Counter Point series published in the Journal of Translational Medicine Craig Slingluff and Cassian Yee engage in a balanced discussion over the advantages and disadvantages of active specific immunization on one side and adoptive transfer of tumor antigen-specific T cells on the other. Future opportunities are presented with special emphasis on the complementarily and biological similarity of the two approaches. It is hoped that lessons learned from either approach will enable better design of future clinical studies combining the simplicity and safety of active-specific immunization with the power of adoptive immune therapy.
